# Synthesis and Assessment of Antimicrobial Composites of Ag Nanoparticles or AgNO_3_ and Egg Shell Membranes

**DOI:** 10.3390/molecules28124654

**Published:** 2023-06-08

**Authors:** Samuel Tomi Aina, Hilda Dinah Kyomuhimbo, Shatish Ramjee, Barend Du Plessis, Vuyo Mjimba, Ali Maged, Nils Haneklaus, Hendrik Gideon Brink

**Affiliations:** 1 Department of Chemical Engineering, University of Pretoria, Pretoria 0002, South Africa; samuel.aina@tuks.co.za (S.T.A.); u21830658@tuks.co.za (H.D.K.); shatish.ramjee@up.ac.za (S.R.); barend.duplessis@up.ac.za (B.D.P.); 2 Human Sciences Research Council, Pretoria 0083, South Africa; vmjimba@hsrc.ac.za; 3 Geology Department, Faculty of Science, Suez University, El-Salam City P.O. Box 43518, Egypt; ali.maged@suezuni.edu.eg; 4 Td Lab Sustainable Mineral Resources, University for Continuing Education Krems, Dr.-Karl-Dorrek-Straße 30, 3500 Krems, Austria; nils.haneklaus@donau-uni.ac.at

**Keywords:** composites, silver nanoparticles, eggshell membrane, adsorption, characterization

## Abstract

Engineering research has been expanded by the advent of material fusion, which has led to the development of composites that are more reliable and cost-effective. This investigation aims to utilise this concept to promote a circular economy by maximizing the adsorption of silver nanoparticles and silver nitrate onto recycled chicken eggshell membranes, resulting in optimized antimicrobial silver/eggshell membrane composites. The pH, time, concentration, and adsorption temperatures were optimized. It was confirmed that these composites were excellent candidates for use in antimicrobial applications. The silver nanoparticles were produced through chemical synthesis using sodium borohydride as a reducing agent and through adsorption/surface reduction of silver nitrate on eggshell membranes. The composites were thoroughly characterized by various techniques, including spectrophotometry, atomic absorption spectrometry, scanning electron microscopy, transmission electron microscopy, Fourier transform infrared spectroscopy, and X-ray photoelectron spectroscopy, as well as agar well diffusion and MTT assay. The results indicate that silver/eggshell membrane composites with excellent antimicrobial properties were produced using both silver nanoparticles and silver nitrate at a pH of 6, 25 °C, and after 48 h of agitation. These materials exhibited remarkable antimicrobial activity against *Pseudomonas aeruginosa* and *Bacillus subtilis*, resulting in 27.77% and 15.34% cell death, respectively.

## 1. Introduction

Composites are now a highly regarded material due to their ability to combine two or more distinct materials, creating a superior product that embodies the strengths of each constituent material [1,2,3]. To further enhance the benefits of these materials, engineers and scientists seek to minimize the drawbacks of each material during the fusion process, resulting in a final product that optimizes the advantages of the individual materials [1,4,5]. Consequently, composites have enabled the engineering of materials with tailored physical, microstructural, chemical, and mechanical properties, making them ideal for a broad range of applications [1,4,6].

Metal nanoparticles possess unique physicochemical properties due to their surface area-to-volume ratios and shapes, making them highly sought after for their antimicrobial, anti-cancer, chemical stability, electronic, magnetic, catalytic, and optical properties [7,8,9]. There are several types of metal nanoparticles currently available, including mercury, iron, gold, cerium, silver, platinum, and thallium. Among these, silver nanoparticles (AgNPs) are the most extensively researched and utilized in various applications, owing to their desirable characteristics such as a high surface area-to-volume ratio, exceptional surface plasmon resonance, ease of functionalization and modification, potent toxicity against pathogens and cancer cells, and catalytic capabilities [10,11,12,13,14,15,16].

Silver nanoparticles (AgNPs) have been observed to occur in different shapes, including oval, spherical, cubic, cylindrical, and triangular, which are influenced by the synthesis method employed [6,17]. There are various methods of synthesizing AgNPs, including physical, chemical, and green techniques. The chemical approach involves the use of reducing agents and stabilizers such as formaldehyde, hydrazine, and sodium borohydride [7].

Over the past decade, the South African poultry industry has continued to enjoy consistent growth. Statistic has revealed that domestic chickens (*Gallus gallus domesticus*) generated sixty-two million tons of eggs in 2008, and 10 years later, this figure has grown to 76.7 million metric tons [18,19]. With a shared mix of 452,000 tons of eggs in 2018 and a 10.2% shell content, the management of this domestic waste now raises concern [20].

Eggshells are characterized by their oval, porous, bioceramic, and calcareous nature. Chicken eggs possess adequate strength to resist physical and pathogenic attacks while also facilitating the exchange of water and gases, which are critical for embryo development [21,22,23]. The nutritional profile of eggshells is complex, with approximately 70% amino acids and several polysaccharides. Some of the proteins found in eggshells are ovalbumin, ovocleidin-17, ovocleidin-116, ovocalyxin-25, ovocalyxin-32, ovocalyxin-36, osteopontin, clusterin, lysozyme, ovo-transferrin, and collagen. The carbohydrates present include uronic acids, sialic acids, chondroitin sulphate A and B, dermatan sulphate, hyaluronic acids, and keratan sulphate [22,24,25]. Of these amino acids, ovocalyxin-36 is particularly noteworthy as it is primarily responsible for the antimicrobial properties of the eggshell [26,27].

According to research [28], the ability of ESM to adsorb metal ions is due to the presence of electrostatic, hydrogen bonding, and van der Waals forces that come into play when ESM is immersed in these ions. Additionally, ESM is a fibrous biomaterial that possesses a high surface area and desirable adhesion ability, making it a suitable candidate for use in composite materials. One particular protein found in ESM, OCX-36, has been identified as having bactericidal properties similar to lipopolysaccharide-binding protein (LBP) and palate, lung, and nasal epithelium clone (PLUNC) proteins [26]. Its ability to resist microbial growth makes it an attractive component for developing antimicrobial materials.

Several studies have demonstrated the utility of composites, eggshells, eggshell membranes, and metal nanoparticles. For instance, Shin et al., Hayajneh et al., and Dwivedi et al. utilized eggshells to produce green aluminum metal composites with exceptional microstructural, tribological, physical, and mechanical properties when compared to pure aluminum, as reported in [29,30,31]. Additionally, an eggshell–rubber composite has been shown to surpass natural rubber in terms of maximum torque, Young’s modulus, and elongation, as discussed in [32]. In [33], a composite of eggshell membrane-templated gold nanoparticles was employed to detect thiabendazole pesticides in Oolong tea.

ES and ESM have been utilized in construction applications, including full or partial replacement of fine aggregate, masonry, production of lightweight foamed concrete, aggregate stabilization, and power insulation [34,35,36,37,38,39,40,41]. Additionally, these materials have been employed in renewable energy as a catalyst for palm kernel biodiesel production [42,43,44] and an oxidizing agent for volatile organic compounds [45]. Moreover, eggshells and their membrane have been utilized in conjunction with osmosis, adsorption, precipitation, photodegradation, and electrodialysis, among other techniques, to decontaminate water bodies polluted with metals and non-metals. ESM has been found to have a high affinity for certain metals such as silver and mercury [46,47,48,49,50].

Given the constant drive for progress and change within the field of materials science, the combination of materials will continue to be significant. Consequently, this study proposes a novel ESM/Nanosilver antimicrobial composite. This research also aims to enhance the adsorption efficiency of chemically produced AgNPs and AgNO_3_ by utilizing the eggshell membrane.

## 2. Results and Discussion

### 2.1. Synthesis and Characterization of AgNPs

**Figure 1:**
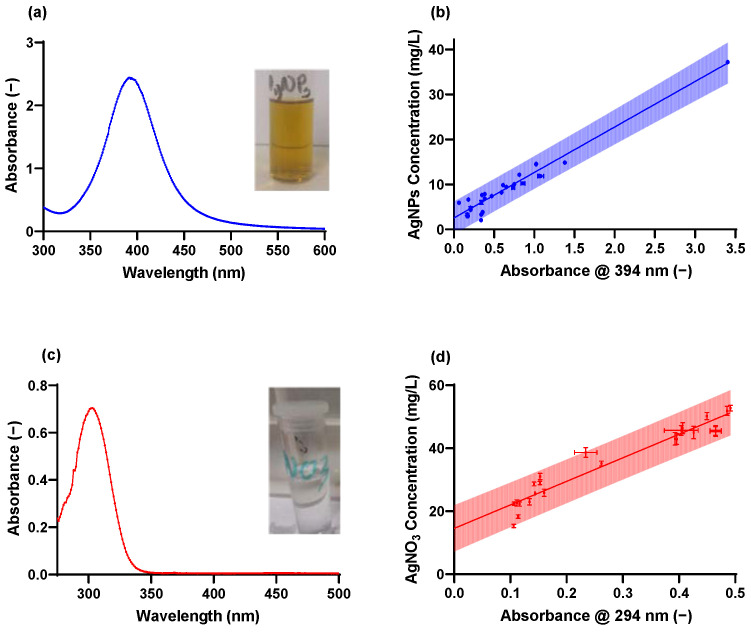
(**a**) Synthesized AgNPs and UV–vis absorbance spectrometry of AgNPs, (**b**) UV–vis absorbance spectrometry of synthesized AgNPs solution, (**c**) The dissolved AgNO_3_ before adsorption and UV-Vis adsorption spectrum of the AgNO_3_, (**d**) the UV-Vis adsorption spectrum of the AgNO_3_ prior to adsorption.

The synthesized nanoparticle suspension was light yellow in colour (Figure 1a) and had a conspicuous UV-vis absorbance peak at 390–400 nm, indicating the successful synthesis of AgNPs (Figure 1b). The strong peaks recorded around 390 nm is a function of surface plasmon resonance (SPR) vis-à-vis the absence of particle aggregation [26,51]. The AgNO_3_ resulted in a clear solution (Figure 1c) and demonstrated maximum adsorption at around 300 nm in solution consistent with results from the literature [52,53] (Figure 1d).

**Figure 2:**
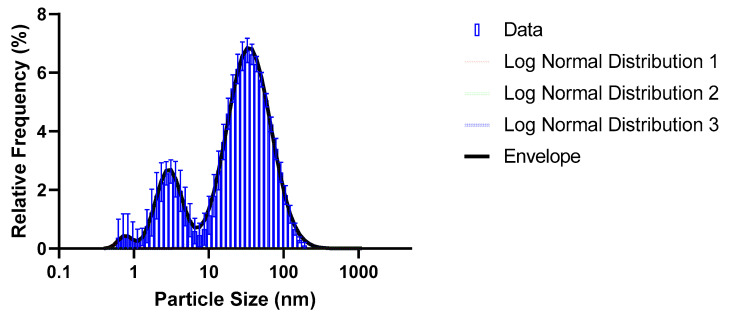
Log-normal distribution for the AgNPs in suspension.

The particle size distribution of the synthesized AgNPs is shown in Figure 2. The data were analysed as per [54], and the results are summarized in Table 1. It can be seen that the AgNPs exhibited a multi-modal distribution with an overall average particles size (*d*_50_) of 29.8 nm (Z-average particles size of 14.3 nm measured by the Zetasizer)—the discrepancy between the *d*_50_ calculated from the distribution curves and the Z-average value relates to the limitations of the Zeta-sizer which requires that the sample be mono-modal, near-spherical particles, in a narrow size distribution [55]. However, TEM imaging of the AgNPs (Figure S4) confirmed that the AgNPs were mostly in sub 50 nm range. According to [56], this particle size makes it suitable for antimicrobial purposes.

### 2.2. Ag/ESM Composite Adsorption Optimization, Kinetics, and Equilibrium Behaviour

The adsorption process was optimized for the mass of ESM required per 10 mL of solution, concentration, agitation time, pH, and temperature. This process was carried out for both AgNPs and AgNO_3_. The slower adsorption of AgNPs compared to AgNO_3_ was evident, possibly because of reduced interactions caused by the uncharged state of the AgNPs.

**Figure 3:**
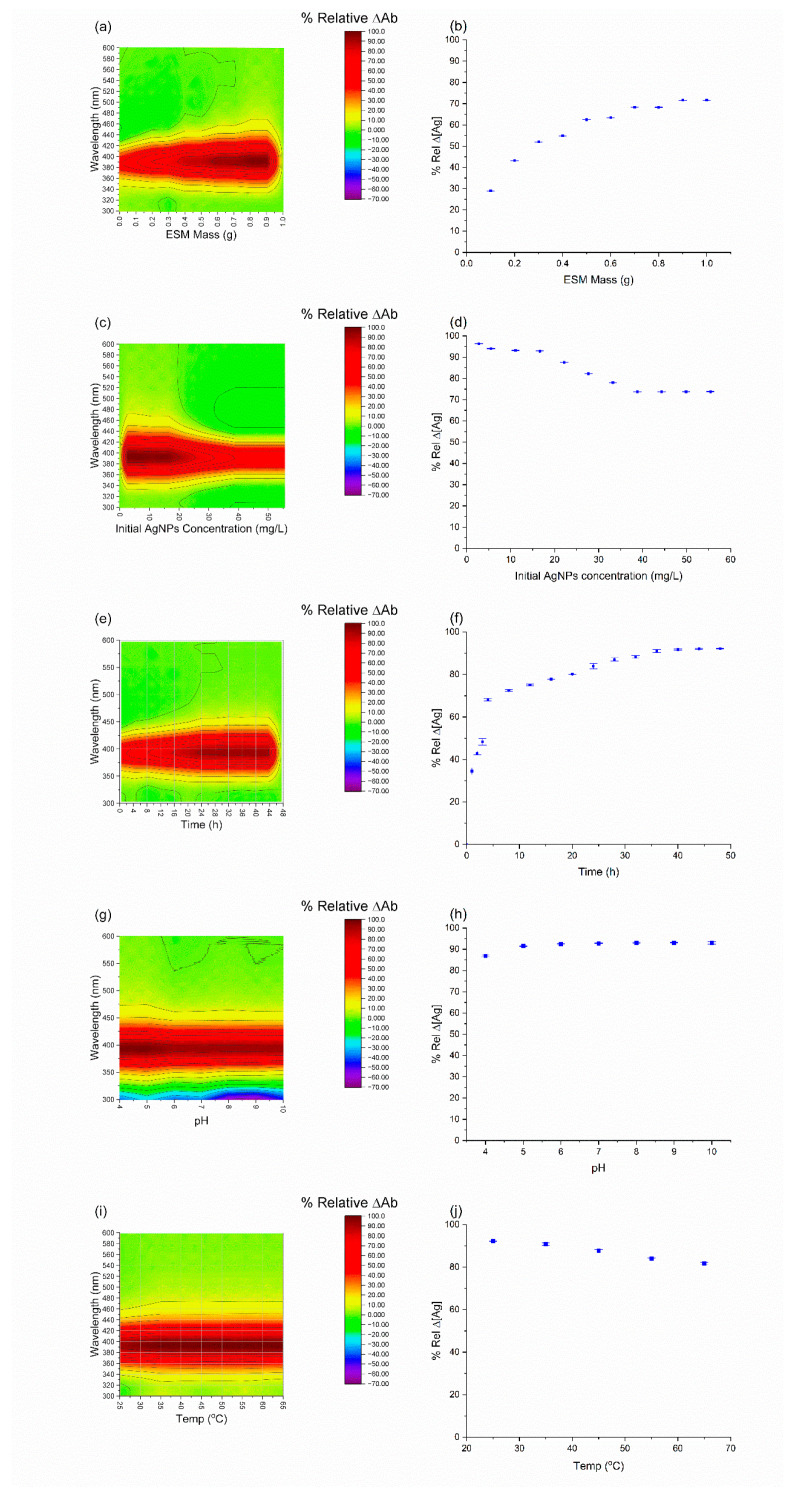
Effect of ESM mass, concentration, initial AgNPs concentration, time, pH, and temperature on AgNPs absorption on ESM at 394 nm. (**a**) Contour plot of UV–vis absorbance spectrometry of AgNO_3_ solution with change in ESM mass. (**b**) Effect of change in ESM mass on absorption. (**c**) Contour plot of UV–vis absorbance spectrometry of AgNPs solution with change in initial AgNPs concentration. (**d**) Effect of change in ESM mass on absorption. (**e**) Contour plot of UV–vis absorbance spectrometry of AgNPs solution with change in agitation time. (**f**) absorption with change in agitation time. (**g**) Contour plot of UV–vis absorbance spectrometry of AgNPs solution with change in pH. (**h**) effect of change in pH on absorption. (**i**) Contour plot of UV–vis absorbance spectrometry of AgNPs solution with change in temperature. (**j**) effect of change in temperature on absorption.

**Figure 4:**
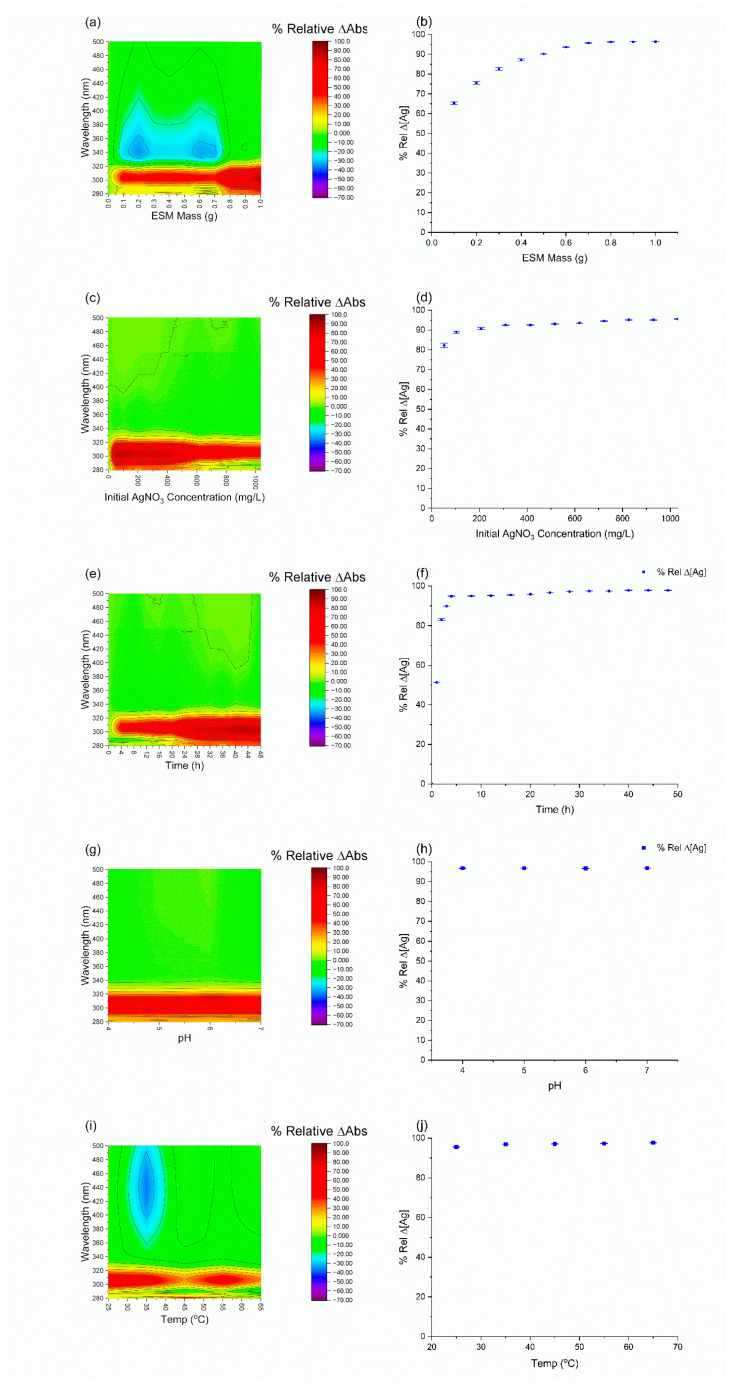
Effect of ESM mass, concentration, initial AgNO_3_ concentration, time, pH, and temperature on AgNO_3_ absorption on ESM at 294 nm. (**a**) Contour plot of UV–vis absorbance spectrometry of AgNO_3_ solution with change in ESM mass. (**b**) Effect of change in ESM mass on absorption. (**c**) Contour plot of UV–vis absorbance spectrometry of AgNO_3_ solution with change in initial AgNO_3_ concentration. (**d**) Effect of change in ESM mass on absorption. (**e**) Contour plot of UV–vis absorbance spectrometry of AgNO_3_ solution with change in agitation time (**f**) absorption with change in agitation time. (**g**) Contour plot of UV–vis absorbance spectrometry of AgNO_3_ solution with change in pH. (**h**) effect of change in pH on absorption. (**i**) Contour plot of UV–vis absorbance spectrometry of AgNO_3_ solution with change in temperature. (**j**) effect of change in temperature on absorption.

AgNPs became lighter in colour with increases in ESM load until 0.7 g, after which the solution became colourless and no longer returned a peak at around 400 nm. This change in colour indicates increased absorption, signified by a reduction in absorbance (Figure 3a). AgNO_3_, on the other hand, was a colourless solution after preparation. However, with every increase in ESM mass, the solution became colloidal with increased absorbance up until 0.4 g (Figure 4a).

As observed in Figure 3c,d, increasing the concentration of AgNPs in solution before absorption decreased the relative Ag removal linearly after absorption. In contrast, the AgNO_3_ saw an increased removal with increased initial concentrations as illustrated in Figure 4c,d.

Figure 3e,f demonstrate that the adsorption of AgNPs initially increased rapidly, with more than 60% of the adsorption taking place within the first 5 h; this was followed by a much slower phase in which the remainder of the adsorption towards equilibrium took until 36 to 48 h. In contrast, the adsorption of AgNO_3_ reached equilibrium within the initial 5 h with negligible further adsorption after this time.

The pH of the stock AgNPs was 6. This was adjusted from 4 to 10, and the effect on absorption was plotted in Figure 3h. The optimization was limited to this range because AgNPs turned dark grey and aggregated at pH less than 4 while it returned no peaks at pH greater than 10. The aggregation at low pH is likely caused by the high concentration of H^+^ ions which lead to the protonation of the AgNP surfaces. The weaker surface charge reduces repulsion, which may lead to aggregation and precipitation. Absorbance and concentration readings were irregular at pH 4 and 5. This is most likely because of how close the solution is to that of the aggregation pH. However, at pH 6 and beyond, the change in pH had no effect on AgNPs absorption. This is indicative of a chemical absorption attributed to the surface charge of the ESM matrix and the ionic strength of the AgNPs [18,47]. The optimal absorption pH range for AgNO_3_ was between 4 and 7. The solution returned no peak below pH 4 and aggregated with a grey colour above pH 7 (likely Ag(OH)). Nevertheless, AgNO_3_ was immune to pH changes, as reported in Figure 4g,h. 

The adsorption temperature was varied from 25 °C to 65 °C. The results (Figure 3i,j) revealed that absorption remained constant after 35 °C. However, the concentration of Ag in the solution increased with an increase in temperature. This signifies the conversion of AgNPs to Ag^+^ at temperatures higher than 25 °C. This is consonant with the know properties of AgNPs and why they are always prepared in an ice bath and stored at cool temperatures [26,57]. AgNO_3_ behaved opposite to the AgNPs, the ESM turned dark brown at 45, 55, and 65 °C, and the concentrations of Ag in solution significantly decreased with increased temperature (Figure 4i,j).

**Figure 5:**
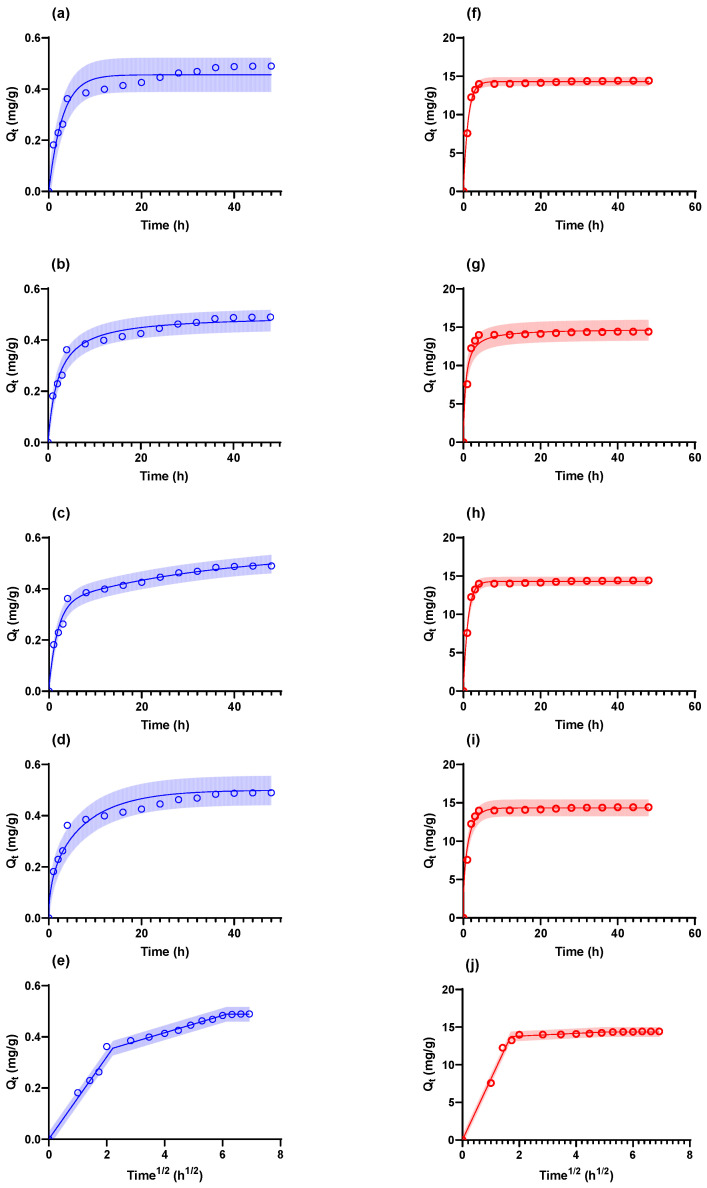
Kinetics of adsorption (**a**–**f**) AgNPs, (**g**–**j**) AgNO_3_. The model fits were (**a**,**f**) PFO, (**b**,**g**) PSO, (**c**,**h**) 2-PA, (**d**,**i**) CIMT, (**e**,**j**) W&M. The shaded areas represent the 95% prediction intervals [70].

It was important to understand the kinetic behaviour of ESM absorption. The kinetic models tested and the corresponding fitted kinetic parameters are summarized in Table 2 and Table 3, respectively. The results of the fittings are shown in Figure 5.

The results for the kinetics model fits demonstrate that the AgNPs adsorption was best described by a 2-PA process with an R^2^ = 0.982 (fast and slow adsorption in parallel [58,59]); this likely indicates heterogeneous interactions between the adsorbate and the adsorbent surface sites [58,59]. The adsorption kinetics of AgNO_3_ is best described by the PFO model (R^2^ = 0.990); this is supported by the observation that the 2-PA model reduces to a PFO model with the same R^2^ value. This means that the system is likely significantly far from the saturation conditions, and therefore, the system behaves effectively irreversibly, and the adsorbate–adsorbent behaviour involves a single surface site per adsorbate molecule [60,61]. The CIMT model predicted an effective diffusivity value (*D_e_*) of 2.78 × 10^−11^ m^2^·s^−1^ for the AgNPs. This does not compare well with results from the literature (D_AgNPs_ = 8.8 × 10^−12^–9.34 × 10^−12^ [62], 9 × 10^−17^–4.67 × 10^−14^ [63]); however, it should be noted that due to the size and characteristics of nanoparticles, these tend to behave more like particles than molecules and, therefore, more readily correspond to Brownian motion predicted by the Einstein–Stokes equation (Equation (1)) [64]:

(1) $$D=\frac{k_{B}.T}{6\pi \eta r}$$

with *D* the diffusion coefficient (m^2^·s^−1^), *k_B_* the Boltzmann constant (1.380649 × 10^−23^ J⋅K^−1^), *T* the absolute temperature (K), *η* the dynamic viscosity (Pa·s), *r* the radius of the particle (m).

**Table 2:** Kinetic models for the adsorption of AgNPs and AgNO_3_ by ESM.

Kinetic Law	Differential Form *	Analytical Form *
Pseudo-first order (PFO) [65]	$\frac{dQ_{t}}{dt}=k_{1}\left(Q_{e}-Q_{t}\right)$	$Q_{t}=Q_{e}\left(1-e^{-k_{1}t}\right)$
Pseudo-second order (PSO) [65]	$\frac{dQ_{t}}{dt}=k_{1}{\left(Q_{e}-Q_{t}\right)}^{2}$	$Q_{t}=\frac{\left(k_{2}Q_{e}^{2}\right)t}{1+k_{2}Q_{e}t}$
Two-phase adsorption (2-PA) [58,59,66,67]	$\frac{dQ_{t,slow}}{dt}=k_{slow}\left(\left(1-\phi \right)Q_{e}-Q_{t,sloe}\right)$	$Q_{t}=Q_{e}\left(\left(1-\phi \right)\left(1-e^{-k_{fast}t}\right)+\phi \left(1-e^{-k_{slow}t}\right)\right)$ [68]
	$\frac{dQ_{t,fast}}{dt}=k_{fast}\left(\phi Q_{e}-Q_{t,fast}\right)$	
	$\frac{dQ_{t}}{dt}=\frac{dQ_{t,slow}}{dt}+\frac{dQ_{t,fast}}{dt}$	
Crank internal mass transfer model (CIMT) [65]	$\frac{\partial Q_{t}}{\partial t}=\frac{D_{e}}{r^{2}}\frac{\partial }{\partial r}\left(\frac{r^{2}\partial Q_{t}}{\partial r}\right)$	$\frac{Q}{Q_{max}}=\{\begin{array}{c} 6{\left(\frac{D_{e}t}{R^{2}}\right)}^{\frac{1}{2}}\left[\pi^{-\frac{1}{2}}-\left(\frac{1}{2}\right){\left(\frac{-D_{e}t}{R^{2}}\right)}^{\frac{1}{2}}\right],\;\;\frac{Q}{Q_{max}}<0.8\\ 1-\frac{6}{\pi^{2}}\exp\left(\frac{-D_{e}\pi^{2}t}{R^{2}}\right),\;\;\frac{Q}{Q_{max}}\geq 0.8 \end{array}$ [68]
Weber and Morris (W&M) [65,69]		$Q_{t}=k_{WM,i}t^{\frac{1}{2}}+C,\;k_{WM,i}=\frac{D_{e,i}}{r^{2}}$ [70]

* The definitions of the kinetic model parameters: General parameters: *Q_t_*—amount of dye adsorbed per unit of adsorbent at time *t* (mg·g^−1^), *Q_e_*—equilibrium adsorption capacity of adsorbent (mg·g^−1^), *r* = the average radius of the adsorbent particles (a conservative estimate of 16 μm was used as all particle diameters < 32 μm). Pseudo-first order (PFO) kinetics: *k*_1_—the PFO rate constant (min^−1^) Pseudo-second order (PSO) kinetics: *k*_2_—the PSO rate constant (g·mg^−1^·min^−1^), Two-phase adsorption (TPA) kinetics: *k_fast_*—the rate constant for the fast TPA adsorption (min^−1^), *k_slow_*—the rate constant for the slow TPA adsorption (min^−1^), *ϕ*—the fraction of adsorption taking place during the fast adsorption step (dimensionless) Crank internal mass transfer kinetic model: *D_e_*—the effective diffusivity of the adsorbate in the system (m^2^·s^−1^) Weber and Morris kinetic model: *K_WM,i_*—the Weber–Morris intra-particle diffusion rate constant for adsorption phase *i* (mg·g^−1^), *D_e,i_*—the effective diffusivity of the adsorbate during adsorption phase *i* (m^2^·s^−1^).

**Table 3:** Fitted kinetic and goodness-of-fit parameters for kinetic models presented in Table 1.

Kinetic Law	AgNPs		AgNO_3_	
	Fitted Parameters	R^2^/RMSE	Fitted Parameters	R^2^/RMSE
PFO	*k*_1_ = 0.339 min^−1^	0.922/0.0316 mg·g^−1^	*k*_1_ = 0.848 min^−1^	0.990/0.261 mg·g^−1^
PSO	*k*_2_ = 0.921 g·mg^−1^·min^−1^	0.970/0.0196 mg·g^−1^	*k*_2_ = 0.113 g·mg^−1^·min^−1^	0.942/0.634 mg·g^−1^
2-PA	*k_fast_* = 0.540 min^−1^	0.982/0.0154 mg·g^−1^	*k_fast_* = *k_slow_* = 0.848 min^−1^ *ϕ* = N/A	0.990/0.261 mg·g^−1^
	*k_slow_* = 0.0297 min^−1^			
	*ϕ* = 0.616			
CIMT	$\frac{D_{e}}{r^{2}}$ = 0.0111 h^−1^	0.940/0.0278 mg·g^−1^	$\frac{D_{e}}{r^{2}}$ = 0.0565 h^−1^ *D_e_* = 1.41 × 10^−10^ m^2^·s^−1^	0.963/0.509 mg·g^−1^
	*D_e_* = 2.78 × 10^−11^ m^2^·s^−1^			
W&M	*D_e_*_1_ = 2.78 × 10^−11^ m^2^·s^−1^	0.986/0.0133 mg·g^−1^	*D_e_*_1_ = 1.41 × 10^−10^ m^2^·s^−1^	0.989/0.276 mg·g^−1^
	*D_e_*_2_ = 1.24 × 10^−12^ m^2^·s^−1^		*D_e_*_2_ = 2.80 × 10^−14^ m^2^·s^−1^	
	*D_e_*_3_ = 0 m^2^·s^−1^		*D_e_*_3_ = 0 m^2^·s^−1^	

For the system under consideration, values for *D* = 1.65 × 10^−11^ m^2^/s for d_p_ = 29.8 nm (the *d*_50_ determined from the particle size distribution—Table 1) and *D* = 3.42 × 10^−11^ m^2^/s (the *Z-average* measured by the Zetasizer—Table 1) were calculated using Equation (1). From these results can be seen that the *D_e_* = 2.78 × 10^−11^ m^2^·s^−1^ falls comfortably within the predicted range, and therefore, the diffusivity of the nanoparticles is not significantly affected by the adsorbent particle characteristics [71]. The effective diffusivity of the AgNO_3_ was determined as *D_e_* = 1.41 × 10^−10^ m^2^·s^−1^_,_ significantly lower than the molecular diffusivity for AgNO_3_ of 1.71 × 10^−9^ m^2^·s^−1^ [72]; this indicates that the AgNO_3_ was significantly limited by the internal structure of the adsorbent. The initial phase of the W&M models corresponded well with the predictions made by the CIMT models for both the AgNPs and AgNO_3_, thereby supporting the observation made for the CIMT model [73].

**Figure 6:**
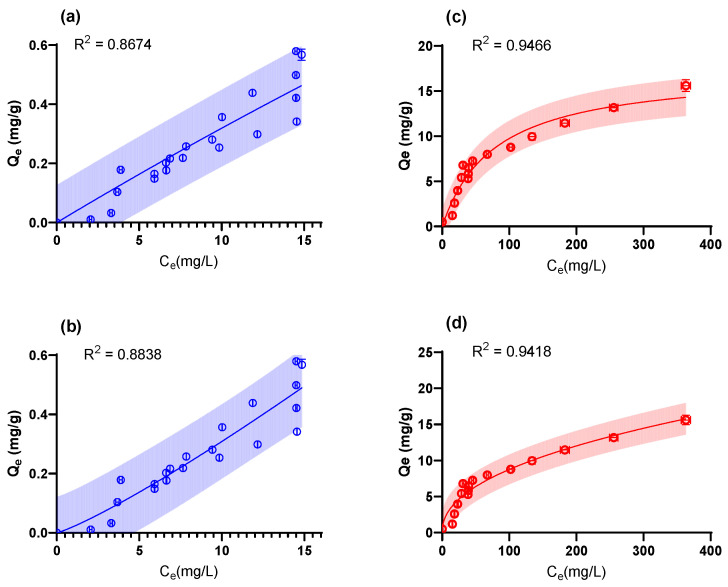
The isotherm model fits for (**a**,**b**) AgNPs and (**c**,**d**) AgNO_3_. The models fitted were (**a**,**c**) Langmuir, (**b**,**d**) Freundlich. The shaded areas represent the 95% prediction intervals.

To elucidate the equilibrium data for the AgNPs and AgNO_3_ adsorption, isotherm models were fitted to the data, as summarized in Table 4. The resulting fitted graphical fits are shown in Figure 6.

The isotherm data for the AgNPs exhibited significant variation, likely because of the variability in the AgNPs sizes (Figure 2) and the heterogeneity of the surface interactions. The data fitted the Freundlich model most closely supporting the heterogenous surface interactions reported for the kinetic data (2-PA model). It is interesting to note that a Freundlich exponent of 0.856 was fitted, indicating an upward-sloping isotherm with increasing adsorption as the concentration rises. These results likely indicate multi-layer adsorption as previously reported for biosorption of Cu-ions by *Pseudomonas syringae* [76].

The isotherm data for the AgNO_3_ appeared to reach an initial maximum at a maximum adsorption capacity of ≈ 17 mg/g. This shape is characteristic of the Langmuir isotherm model in which the surface is saturated with a monolayer of adsorbate (consistent with the observations reported in the kinetic runs).

### 2.3. Characterization of Ag/ESM Composites

**Figure 7:**
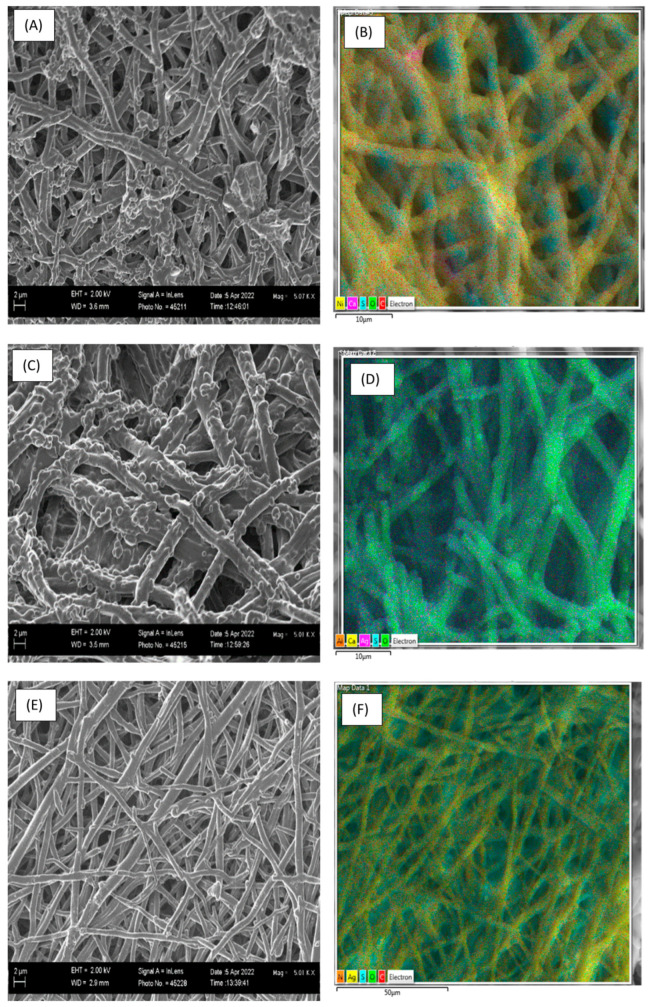
SEM images (**A**,**C**,**E**) EDS maps (**B**,**D**,**F**) of ESM composite. (**A**,**B**) ESM. (**C**,**D**) AgNO_3_/ESM. (**E**,**F**) AgNPs/ESM.

The fibre-like structures of ESM composites were analysed using SEM-EDS (Figure 7). This porous fibril structure responsible for ESM’s absorption capacity [51] proved effective. As observed in the EDS map image in Figure 7d,f (and Supplementary Figures S1 and S2), AgNO_3_ and AgNPs were evenly absorbed on the surface of the membrane. Both Ag solutions were significantly absorbed into the ESM with weighted compositions of 15.68% and 4.87% for AgNO_3_ and AgNPs, respectively (Table 5).

The AgNPs ESM structure shows a much more even distribution of the coated Ag, which dominates the surface composition; this is indicative of a slower, more controlled deposition (Figure 7d and Figure S1). The AgNO_3_ ESM shows nodules on the fibre surface. This suggests compounds are absorbed, producing a rougher texture, with predominantly oxygen on the surface; this is observed via the cyan-like colour as a mixture of green (oxygen) and magenta (silver), as shown in Figure 7f and Figure S2. This suggests the presence of AgO as the form of Ag absorbed. This supports the notion that AgNO_3_ is reduced to AgO as part of the absorption process. This rough surface is likely a product of the high rates of absorption. The absorption occurred on both the ESM matrix as well as previously absorbed material. In this mechanism, the growth of crystallites is favoured over nucleation. This suggests an abundance of absorption sites; the same conclusion is reached through modelling.

All materials exhibited broad FTIR absorption peaks at 3275 and 2927 cm^−1^ corresponding to O-H and N-H groups stretching mode. Peaks of C=O (amide I), CN/NH (amide II), and CN/NH (amide III) modes were prominent at 1638, 1517, and 1442 absorption bands. This corresponds to reports from [77] with similar peaks. The 1235 cm^−1^ is often correlated with the stretching vibration of C-O bonds and interaction with some amino acids. The shifts and/or changes in the intensity of these peaks indicate an interaction of the nanoparticles with the protein structure.

**Figure 8:**
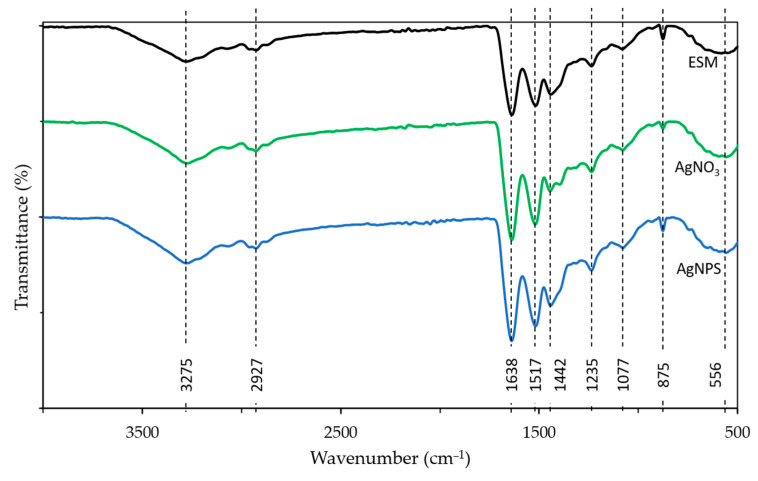
FTIR spectra of ESM, AgNO_3_/ESM, and AgNPs/ESM.

The bands at 1077, 875, and 556 cm^−1^ correspond to the C-O stretching modes, CH_2_ deformation, and unknown peaks in the fingerprint region. The higher intensity of these peaks in the Ag/ESM samples compared to ESM indicates the absorption of AgNO_3_ and AgNPs to ESM. Likewise, peak shifts were identified in these bands between ESM and the composite samples, which were also indicative of the absorption process [77] (Figure 8 and Table 6).

The results from FTIR suggest a strong interaction of the silver compounds with the amine groups present in the eggshell membrane. The interaction with the proteins may have led to the absorption of nanoparticles to the surface, likely through van der Waals forces. Similarly, proteins (glycine, lysine, or cysteine) may have contributed to the reduction of AgNO_3_ to AgO.

**Figure 9:**
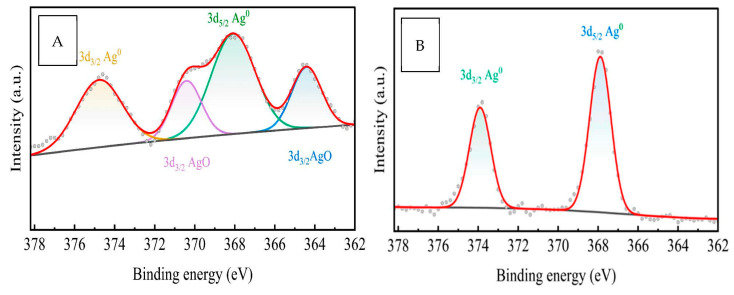
XPS spectrum for (**A**) AgNO_3_/ESM and (**B**) AgNPs/ESM.

X-ray photoelectron spectroscopy (XPS) was used to investigate the valence state and chemical composition of Ag absorbed on ESM, as seen in Figure 9 and Table 3. Elemental signals of O1s, C1s, N1s, and CaCO_3_ compounds were detected on ESM, AgNO_3_/ESM, and AgNPs/ESM samples. The ESM sample had no trace of silver, as expected. AgNO_3_/ESM sample had signals of both elemental silver (3d_5/2_ Ag0 and 3d_3/2_ Ag^0^ with peaks at 368.08 and 374.77, respectively) and silver oxide (3d_3/2_ AgO with peaks at 364.41 and 370.41). AgNPs/ESM, on the other hand, only had signals of elemental silver (3d_5/2_ Ag^0^ and 3d_3/2_ Ag^0^ with peaks at 367.89 and 373.91, respectively) with similar peaks as those found in the AgNO_3_/ESM composite. These AgNPs peaks are similar to those already reported by [78], signifying the successful absorption of AgNPs on ESM. The similar Ag^0^ peaks found in both AgNO_3_/ESM and AgNPs/ESM also signify the reduction of AgNO_3_ to nanoparticles by ESM during the adsorption process.

The results indicate that the adsorption mechanism is dictated by surface interactions between the AgNPs/AgNO_3_ and the ESM. According to Xin et al. [79], the interactions of heavy metals and ESM is strongly affected by surface species. In contrast, however, the lack of pH effects on either AgNPs or AgNO_3_ adsorption negates the likelihood of significant electrostatic or ion exchange mechanisms present in the Ag/ESM adsorption—as proposed by Xin et al. [79]. It is clear from the shifts in FTIR peaks (Table 7) that significant interactions between the Ag species and the O-H, NH, C=O (amide I), CN/NH (amide II), and CN/NH (amide III). C-O bond, C-O stretching, and CH_2_ deformation with significant shifts observed. This is confirmed by the Ag distribution observed in the SEM-EDS (Figure 7) and the significant shifts in the O1s and N1s peaks in the XPS results (Table 7).

### 2.4. Antimicrobial Activity of Ag/ESM Composites

*P. aeruginosa* is an opportunistic gram-negative pathogen that is common and highly adaptive to various environmental conditions, including aquatic environments [80]. It is highly resistant to antimicrobials, with a percentage resistance of 33.9%. This makes it vital to treat concrete, especially in aquatic environments [81,82]. On the other hand, *B. subtilis,* though gram-positive, can survive harsh conditions such as high temperature, UV, and ɣ-radiation. It is also found in various environments and broadly adapted to grow in diverse settings within the biosphere, from soil to marine habitats [83,84]. *B. subtilis* has shown the highest occurrence in hospital settings compared to other bacteria groups, with the biggest percentage of multiple resistant strains to antimicrobials. Their ability to survive harsh conditions makes them a problem for cleaning and disinfection [85].

**Figure 10:**
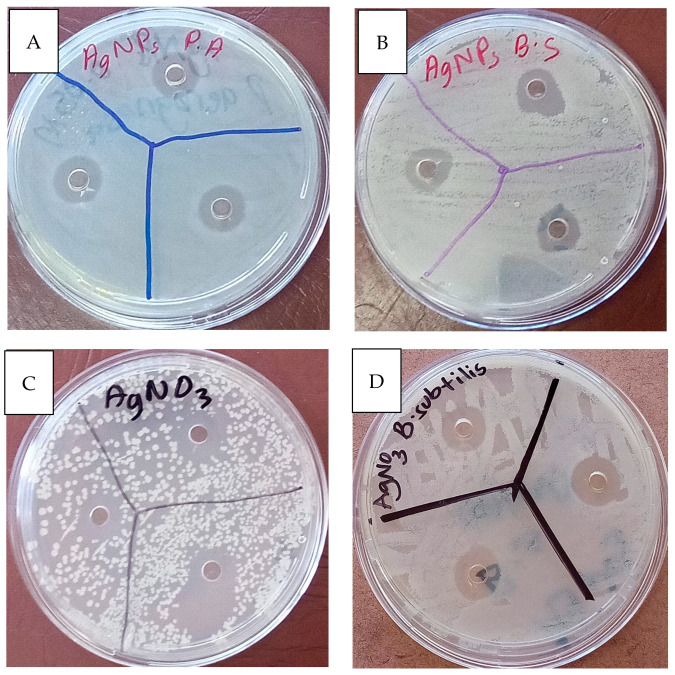
Agar well diffusion images. (**A**) AgNPs with *P. aeruginosa*, (**B**) AgNPs with *B. subtilis*, (**C**) AgNO_3_ with *P. aeruginosa*, (**D**) AgNO_3_ with *B. subtilis*.

**Figure 11:**
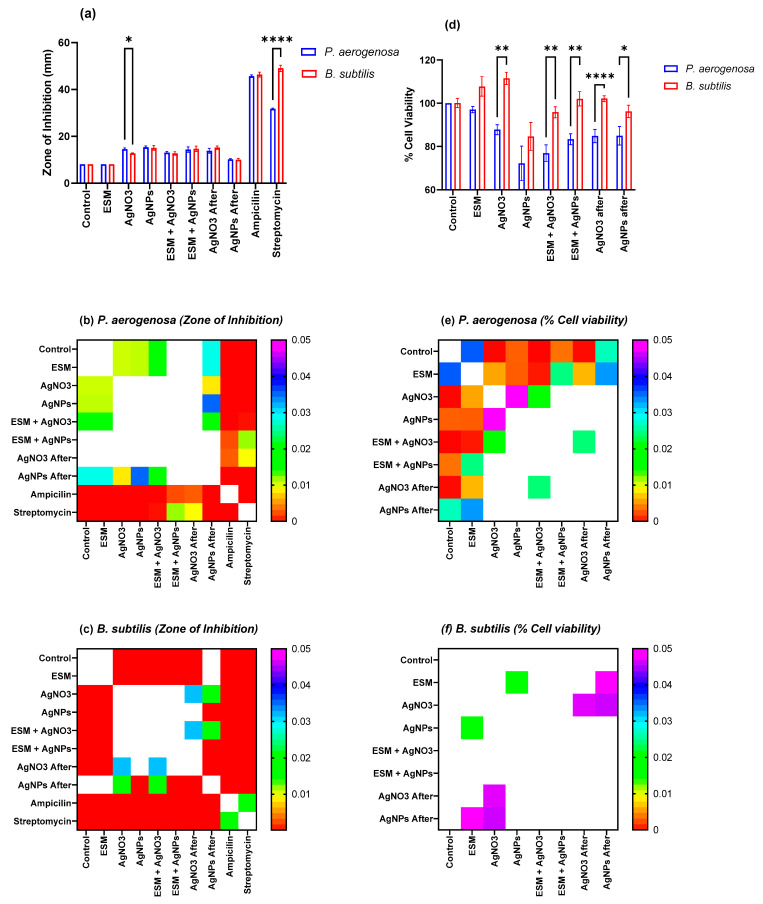
(**a**,**d**) Results of Zone of Inhibition (**a**) and % Cell Viability (**d**) results for the different microbial species exposed to the different combinations of solid materials. The brackets indicate those comparisons between the species that exhibited significant differences, the extent of the significance was indicated as: * 0.033 < *p* < 0.05, ** 0.033 < *p* < 0.021, **** *p* < 0.0002. (**b**,**c**,**e**,**f**) Show heatmaps of the adjusted *p*-values when comparing the ZoI and % Cell viability results for the various materials. White indicates adjusted *p*-values > 0.05 (i.e., no significant difference at the 95% confidence level). (**b**,**e**) for *P. aerogenosa* and (**c**,**f**) for *B. subtilis*.

For purposes of consistency, concentrations were based on the concentration of Ag in the sample, and the mass of ESM was determined from the mass that could provide 100 µg/L of AgNPs. The agar well diffusion showed clear halos of varying diameters, indicating antimicrobial properties for AgNPs, AgNPs/ESM, and AgNO_3_, as shown in Figure 10. This is evidenced by both bacteria and the strength of the antimicrobial activity depending on the component tested. These results were fitted into the zone of inhibition bar charts, as shown in Figure 11.

The positive controls, streptomycin and ampicillin, gave extremely large zones as compared to the AgNPs-ESM, indicating that the bacteria were more susceptible to their antimicrobial activity. ESM showed no effect on the growth of either *B. subtilis* or *P. aeruginosa*, implying that all the antimicrobial activity observed in the Ag-ESM was due to the adsorbed AgNPs on the ESM.

According to Figure 11a, the results of the zone of the inhibition tests presented show significant inhibition to the growth of both *P. aeruginosa* and *B. subtilis*. This revealed that ESM alone had the least effect on either bacterium, while AgNPs were the most effective, followed by AgNO_3_ excluding the antibiotics. This observation could be the result of the reduction of AgNO_3_ to AgNPs during absorption. AgNO_3_, after adsorption, had the most antibacterial effect. As expected, the AgNPs medium after adsorption had the least significant because most of its antimicrobial particles have been absorbed into the ESM surface.

When the components were exposed to bacteria in a liquid medium, the MTT assay showed that *P. aeruginosa* is more susceptible to the antimicrobial activity of AgNPs-ESM compared to *B. subtilis* (Figure 11d). *P. aeruginosa* showed a percentage cell death of 27.77% and 23.16% for AgNPs and AgNPs-ESM, respectively. In contrast, it is evident that 10 µM AgNO_3_, ESM, and AgNO_3_-ESM had no significant cytotoxic effect on *B. subtilis* while AgNPs and AgNPs-ESM showed minimal antimicrobial activity with 15.34% and 4.15% cell death for AgNPs and AgNPs-ESM, respectively, when compared to the control samples.

The results were supported by Tukey multiple comparison tests [86], which assessed the significance of the differences in the observations for ZOI and MTT for *B. subtilis* and *P. aeruginosa.* The results are summarized in Figure 11b,c,e,f, and show that when agar-well diffusion was used, there was no observable difference between the antimicrobial activity of AgNPs-ESM against *B. subtilis* and *P. aeruginosa*—except for a small but significant difference in the AgNO_3_ only well, and a much more significant effect of Streptomycin on *P. aeruginosa* (It has been observed that *P. aerogenosa* exhibits intrinsic antibiotic resistance to Streptomycin [87]). In contrast, marked differences in the effects on *B. subtilis* and *P. aeruginosa* were observed in the MTT tests, with *B. subtilis* exhibiting significant tolerance to metabolic inhibition while *P. aerogenosa* was significantly inhibited.

Based on the results, AgNO_3_ and AgNPs produces ESM composites with good antimicrobial properties and can be used for further applications such as wound healing and incorporation into antimicrobial concrete.

## 3. Materials and Methods

### 3.1. Preparation of Eggshell Membranes (ESM)

Eggshells were sourced from eateries within the University of Pretoria and its surroundings. Within 24 h of collection, all shells were washed and dried at 60 °C for 60 min. Decontaminated shells were stored in plastic bags until separation. To aid membrane separation, the shell was soaked in 1 mol/L acetic acid for 17 min, and thereafter, the ESM was removed from the shell by hand [88]. All separated membranes were washed in deionized water, dried, and stored.

### 3.2. Synthesis of AgNPs

AgNPs were synthesized by chemical reduction of AgNO_3_ with sodium borohydride (NaBH_4_) as well as by direct membrane reduction.

The method described in [26] was employed to carry out the chemical reduction. NaBH_4_ functioned as a reducing agent, while trisodium citrate dihydrate (Na_3_C_6_H_5_O_7_·2H_2_O) was used as a ligand. Initially, 100 mL of Na_3_C_6_H_5_O_7_·2H_2_O solution (0.01 M), 50 mL silver nitrate (AgNO_3_) solution (0.01 M), and 50 mL 0.01 M NaBH_4_ were prepared in deionized water. Then, 20 mL of deionized water, 1 mL of AgNO_3_, and 1 mL of Na_3_C_6_H_5_O_7_·2H_2_O from the prepared solution were added to a 100 mL beaker placed in an ice bath. To prepare a nanosilver solution, the AgNO_3_-Na_3_C_6_H_5_O_7_ solution was stirred for 5 min using a magnetic stirrer, and then 1.2 mL of NaBH_4_ was added dropwise until the colour of suspension changed to bright yellow. The reactor was stirred continuously for two hours to ensure a complete reduction reaction.

### 3.3. Detection and Characterization of AgNPs

The synthesized silver nanoparticles were characterized using a spectrophotometer, atomic absorption spectrometry (AA), scanning electron microscope (SEM), transmission electron microscopy (TEM), and Fourier transform infrared spectroscopy (FTIR).

The UV-vis absorbance spectra of the nanoparticles were measured from 300 nm to 600 nm with the use of the VWR UV-1600PC spectrophotometer. The Zetasizer Nano-ZS90 instrument (Malvern Instruments, Malvern, UK) was used for particle-size analysis.

An atomic absorption spectrometer (Perkin Elmer AAnalyst 400, Waltham, MA, USA) was used to measure silver concentration with an SJ hollow silver lamp. AgNO_3_ was used to generate the standard linear calibration curve. The samples were diluted with Distilled-deionized water to the Ag concentration within the instrument’s 1 mg/L linear range. The analysis was conducted in triplicates.

### 3.4. Production of ESM Composites

Both synthesized AgNPs and AgNO_3_ were adsorbed into ESM. Dried ESM of particle sizes ranging from 1 mm to 5 mm were placed in 40 mL glass Polytops containing double diluted AgNPs or AgNO_3_ and agitated in an oscillator for adsorption to take place. The process was optimized using UV-vis absorbance spectra and AA analysis for pH (6.0–9.0), reaction time (30 min–48 h), temperature (25–65 °C), and concentration. pH was adjusted using HNO_3_ (0.1 M) or NaOH (0.1 M). The concentration range for the AgNPs was limited by the concentration AgNPs synthesized using the method described in Section 3.2, i.e., 35.5 mg/L. The concentration range of AgNO_3_ was limited by the solubility limit of AgNO_3_ in water.

Transmission electron microscopy (TEM) images were obtained for post-adsorption nanoparticle size determination. The Jeol 2100F FEG TEM with an EDS detector and an accelerating voltage of 30 kV was used.

### 3.5. Characterization of ESM Composites

The morphology of the membranes before and after adsorption of the nanoparticles was studied in a Zeiss Ultra PLUS FEG scanning electron microscope (SEM). Samples were dried before being sputter-coated with carbon in a Quorum Q150T ES coater for imaging. SEM energy dispersive X-ray analysis (EDX) was also conducted to understand the distribution and elemental composition of the nanoparticles on the membranes.

The Bruker ALPHA II Compact FT-IR Spectrometer using the OPUS 7.0 software was employed to determine all functional groups likely to be found in the ESM/AgNO_3_ composite. All samples were dried and grounded prior to the analysis. The chemical state of Ag absorbed on ESM was analyzed using X-ray photoelectron spectroscopy (XPS, Thermo ESCAlab 250Xi, Monochromatic Al kα, 300W, Thermo Fisher Scientific, Waltham, MA, USA).

### 3.6. Antimicrobial Activity of AgNPs, AgNO_3_, and ESM Composites

The antimicrobial activities against *Bacillus subtilis* and *Pseudomonas aeruginosa* were determined using the agar well diffusion method and MTT assay. For agar well diffusion, 8 mm diameter holes were punched in agar plates containing the desired bacteria and filled with 100 µL of 100 µg/L of AgNPs, AgNO_3_, AgNPs/ESM, and AgNO_3_/ESM solution. Note the AgNPs/ESM and AgNO_3_/ESM were the composites obtained at the maximum adsorption capacities, i.e., *circa* 0.6 mg/g and *circa* 16 mg/g, respectively. The agar plates were then incubated at 37 °C for 24 h. Streptomycin and ampicillin were used as positive controls for *B. subtilis* and *P. aeruginosa,* respectively. Distilled water was used as a negative control.

MTT (3-(4,5-dimethylthiazolyl-2)-2,5-diphenyltetrazolium bromide) assay is a dependable and responsive colorimetric assay used to evaluate the metabolic activity of cells. This tetrazolium dye can be reduced into a purple-coloured insoluble compound known as formazan by specific bacterium enzymes. The concentration of formazan is determined by measuring its absorbance using a spectrometer within the 500 to 700 nm range. As the number of viable bacteria increases, the concentration of formazan also increases, resulting in a more intense purple colour and a higher absorbance value [89,90,91]. Overnight cultures of bacteria were grown in nutrient broth at 37 °C while shaking at 150 rpm until an optical density of 0.4 was obtained. The cultures were centrifuged, the supernatant was washed twice with distilled water and resuspended in distilled water, and optical density was adjusted to ~1. Exposures of bacteria (OD600 = 0.2) to AgNPs, AgNO_3_, AgNPs/ESM, AgNO_3_/ESM, ESM, residual AgNO_3_ solution after ESM adsorption and residual AgNPs solution after ESM adsorption was carried out in 96 well plates at a concentration 10 µL while shaking at 85 rpm for 3 h. 10 µL of MTT solution was added to 90 µL of exposed bacteria and incubated for 1 h in a thermostatic shaker at 37 °C for 200 rpm in the dark. 100 µL of DMSO was then added to the mixture and incubated at room temperature for 1 h in the dark while shaking at 70 rpm. The absorbance was measured at 560 and 700 nm using a microplate reader. A stock solution of 5 mg/mL of MTT in 0.1 M PBS (pH 7) was used.

## 4. Conclusions

ESM has proven to be a great absorption agent for both AgNO_3_ and AgNPs. This is because of its fiber-like structure, as observed through SEM micrographs, the result obtained by [88]. Absorption was optimum at 70% concentration, pH 6, after 48 h of agitation time and at room temperature. The strong AgNP peaks recorded at 394 nm is a function of surface plasmon resonance (SPR) vis-à-vis the absence of particle aggregation. These observations are inconsonant with the findings. Both AgNPs and AgNO_3_ were evenly absorbed on ESM as observed by EDX maps, with 15.68% and 4.87% composition for AgNO_3_ and AgNPs, respectively.

During the adsorption of AgNO_3_, an increase in several FTIR peaks was observed. This was thought to be an indication of the reduction of AgNO_3_ to Ag nanoparticles by the ESM. This assumption was later confirmed by the result of XPS analysis and the zone of inhibition test of AgNO_3_ after on *B. subtilis*. XPS results, in conjunction with other microstructural analyses, revealed that ESM was able to synthesize Ag NPs from AgNO_3_ during adsorption.

This optimized composite is deemed to find good use in antimicrobial applications in the medical and construction fields. With respect to the availability of ESM and the continuous advocacy for a circular economy, Ag/ES M composites will be a good step toward achieving a waste reduction in the food industry.

## Figures and Tables

**Table 1:** Distribution properties of the particle-size distribution of synthesised AgNPs (Figure 2).

Distribution 1 (Red Peak)		Distribution 2 (Green Peak)		Distribution 3 (Blue Peak)		Total Distribution (Dashed Line/Bars)	
% of Total	*d*_50_ (nm) Predicted ^1^	% of Total	*d*_50_ (nm) Predicted ^1^	% of Total	*d*_50_ (nm) Predicted ^1^	*d*_50_ (nm) Predicted ^1^	*Z*-Average (nm) Measured ^2^
1.62	0.82	18.7	3.15	79.4	37.1	29.8	14.3

^1^ As predicted from the distribution curves; ^2^ As measured directly by the Zetasizer.

**Table 4:** Isotherm models for the adsorption of AgNPs and AgNO_3_ by ESM.

Isotherm		AgNPs		AgNO_3_	
	Non-Linear Form *	Fitted Parameters	R^2^/RMSE	Fitted Parameters	R^2^/RMSE
Langmuir [65,74,75]	$Q_{e}=\frac{k_{L}Q_{max,L}C_{e}}{1+k_{L}C_{e}},$	$k_{L}=0.0050\;L\cdot {\mathrm{mg}}^{-1}$	0.867/ 0.060 mg·g^−1^	$k_{L}=0.0127\;L\cdot {\mathrm{mg}}^{-1}$	0.947/ 0.935 mg·g^−1^
		$Q_{max}=6.71\;\mathrm{mg}\cdot g^{-1}$		$Q_{max}=17.41\;\mathrm{mg}\cdot g^{-1}$	
Freundlich [75]	$Q_{e}=K_{F}C_{e}^{\frac{1}{n_{F}}}$	$K_{F}=0.0209\;\mathrm{mg}\cdot g^{-1}{\left(L\cdot {\mathrm{mg}}^{-1}\right)}^{\frac{1}{n_{F}}}$	0.884/ 0.067 mg·g^−1^	$K_{F}=1.07\;\mathrm{mg}\cdot g^{-1}{\left(L\cdot {\mathrm{mg}}^{-1}\right)}^{\frac{1}{n_{F}}}$	0.942/ 0.977 mg·g^−1^
		$n_{F}=0.856$		$n_{F}=2.19$	

* The definitions of the isotherm parameters: Langmuir isotherm: *k_L_*—the Langmuir equilibrium constant (L·mg^−1^), *Q_max_*_,*L*_—the Langmuir maximum adsorption capacity (mg·g^−1^); Freundlich isotherm: *K_F_*—the Freundlich intensity parameter ((mg·g^−1^)(L·mg^−1^)^1/nF^), *n_F_*—Freundlich isotherm exponent (dimensionless).

**Table 5:** EDS elemental analysis of ESM composites.

Element	ESM (Wt%)	AgNO_3_/ESM (Wt%)	AgNPs/ESM (Wt%)
Ag	-	15.68	4.87
S	46.42	30.52	36.05
O	49.97	53.19	57.62
Si	0.85	-	-
Ca	2.77	0.61	1.45

**Table 6:** FTIR spectra of ESM composites.

Species	O^_^H	N^_^H	C=O (Amide I)	CN/NH (Amide II)	CN/NH (Amide III)	C^_^O	C^_^O Stretching	CH_2_ Deformation
ESM	3274.36	2926.90	1637.21	1517.58	1439.67	1235.67	1080.33	874.94
AgNO_3_/ESM	3269.37	2927.48	1637.56	1518.81	1444.05	1235.26	1078.68	875.19
AgNPs/ESM	3275.56	2927.09	1638.14	1517.33	1442.24	1235.33	1077.26	875.50

**Table 7:** XPS analysis of ESM composites.

	ESM	AgNO_3_/ESM	AgNPs/ESM
O1s	531.40 and 532.35	531.79 and 533.48	531.32 and 532.15
N1s	399.76	399.86	399.70
3d Ag^0^	-	368.08 and 374.77	367.89 and 373.91
3d AgO	-	364.41 and 370.41	-
Ca 2p	347.22 and 350.80	347.19 and 350.75	347.21 and 350.78
C1s	284.57, 285.50, 287.75, and 288.57	284.56, 285.31, 287.45, and 288.74	284.54, 285.11, 287.5, and 288.4

## Data Availability

The data presented in this study are available on request from the corresponding author.

## Supplementary Materials

The following supporting information can be downloaded at: https://www.mdpi.com/article/10.3390/molecules28124654/s1, Figure S1: ESM/AgNO_3_ EDS Map; Figure S2: ESM/AgNO_3_ EDS Map; Figure S3: ESM EDS Map; Figure S4: TEM image of particles AgNPs particles at different magnifications.
