# Supplementary material for: Synthesis and Assessment of Antimicrobial Composites of Ag Nanoparticles or AgNO3 and Egg Shell Membranes
Source: Molecules. 2023 Jun 8;28(12):4654. doi: 10.3390/molecules28124654 (PMC10304066; doi:10.3390/molecules28124654)
Supplement: Supplementary file 1 [file molecules-28-04654-s001.zip › molecules-2425757-supplementary.pdf]

Supplementary Material

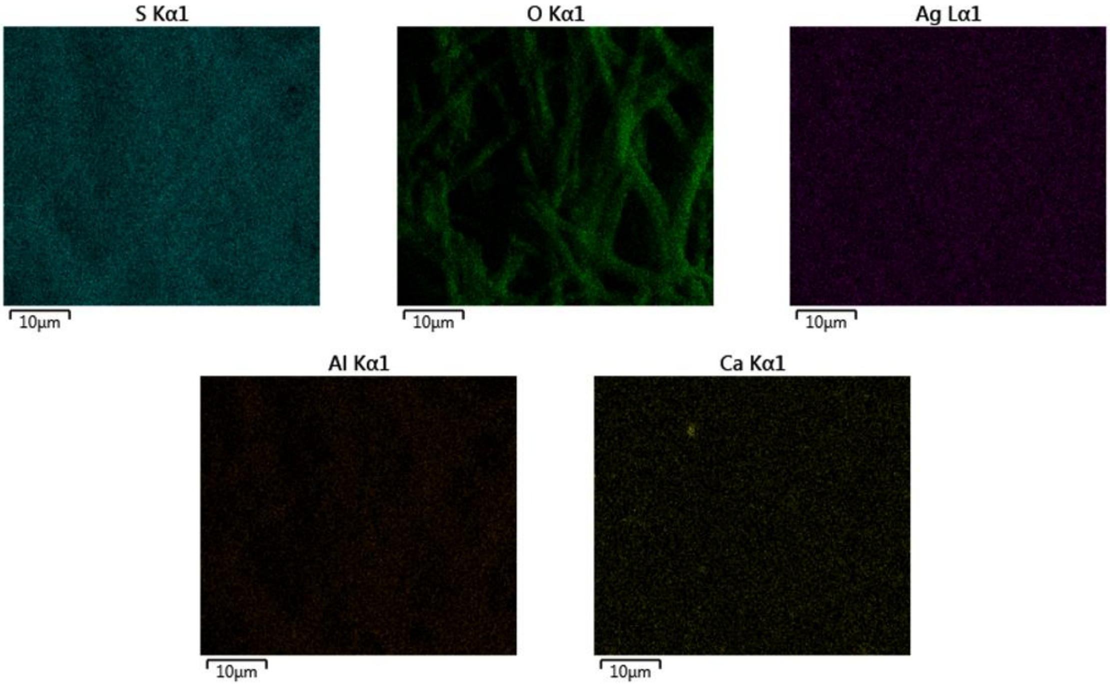

Figure **S1** ESM/$AgNO_3$ EDS Map

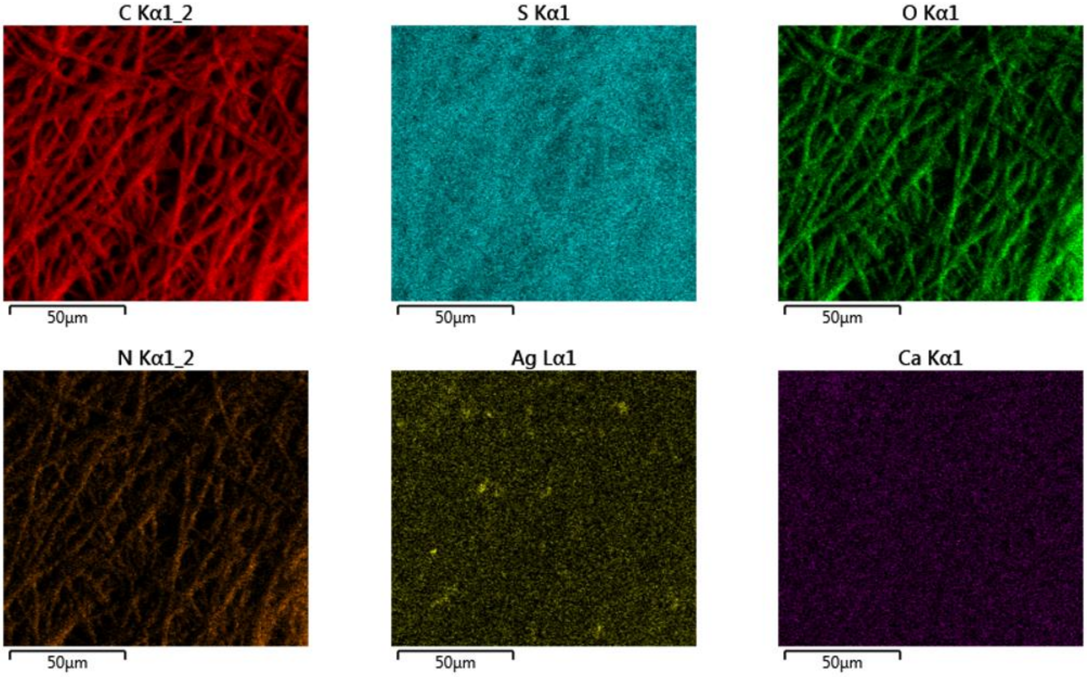

Figure S2 ESM/AgNPs EDS Map

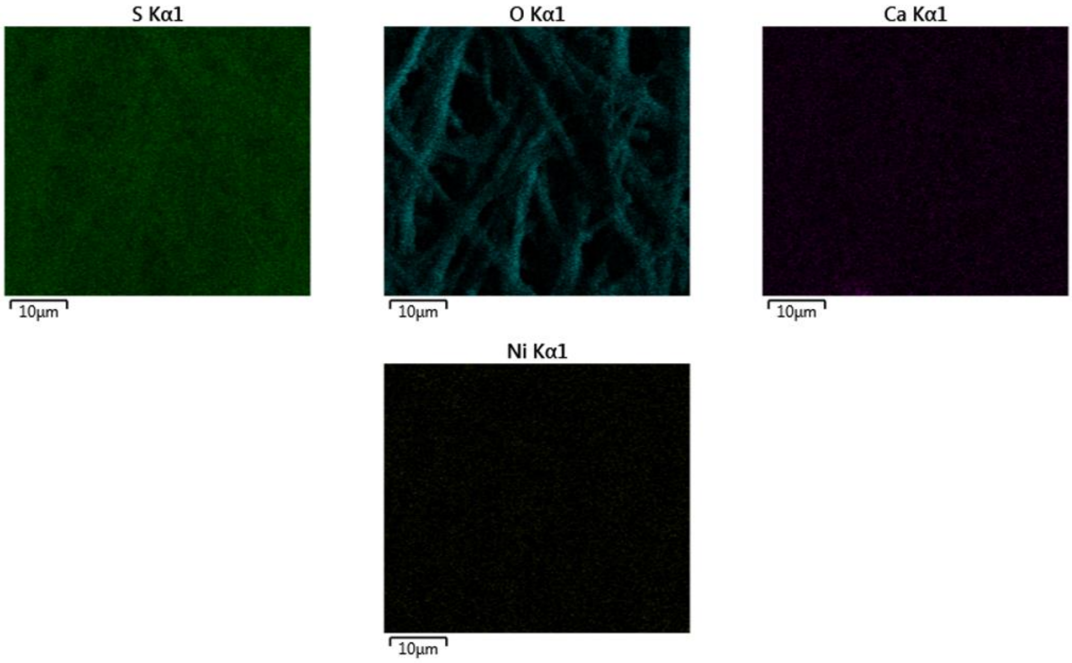

Figure S3 ESM EDS Map

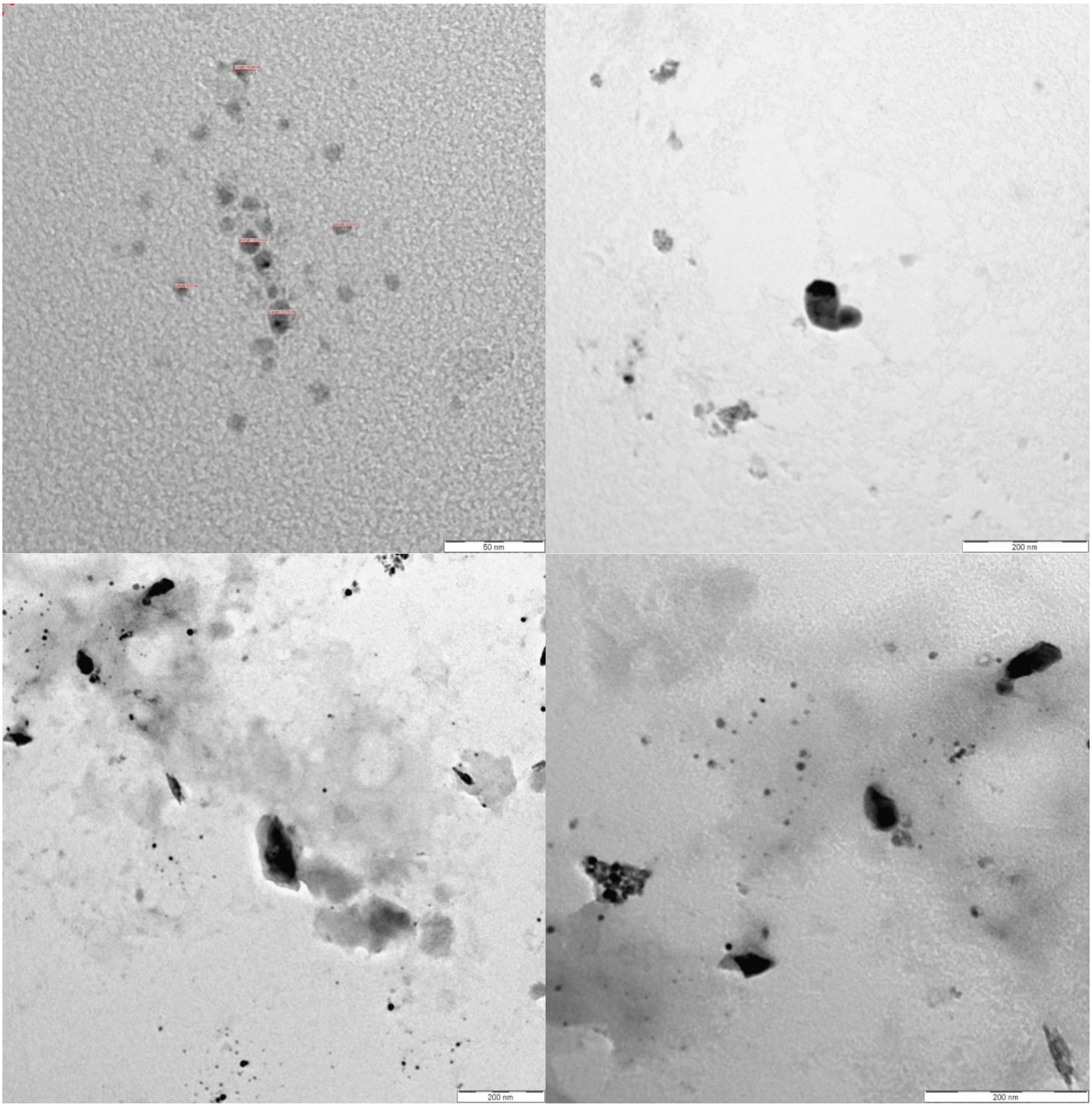

Figure S4 TEM image of particles AgNPs particles at different magnifications (see scale bar)
